# The Role of the PTEN Tumor Suppressor Gene and Its Anti-Angiogenic Activity in Melanoma and Other Cancers

**DOI:** 10.3390/molecules29030721

**Published:** 2024-02-04

**Authors:** Jacqueline Maphutha, Danielle Twilley, Namrita Lall

**Affiliations:** 1 Department of Plant and Soil Sciences, University of Pretoria, Pretoria 0002, South Africa; 2 School of Natural Resources, University of Missouri, Columbia, MO 65211, USA; 3 College of Pharmacy, JSS Academy of Higher Education and Research, Mysuru 570015, India

**Keywords:** cancer, melanoma, angiogenesis, vascular endothelial growth factor, phosphatase tensin homolog, nanocarrier formulations

## Abstract

Human malignant melanoma and other solid cancers are largely driven by the inactivation of tumor suppressor genes and angiogenesis. Conventional treatments for cancer (surgery, radiation therapy, and chemotherapy) are employed as first-line treatments for solid cancers but are often ineffective as monotherapies due to resistance and toxicity. Thus, targeted therapies, such as bevacizumab, which targets vascular endothelial growth factor, have been approved by the US Food and Drug Administration (FDA) as angiogenesis inhibitors. The downregulation of the tumor suppressor, phosphatase tensin homolog (PTEN), occurs in 30–40% of human malignant melanomas, thereby elucidating the importance of the upregulation of PTEN activity. Phosphatase tensin homolog (PTEN) is modulated at the transcriptional, translational, and post-translational levels and regulates key signaling pathways such as the phosphoinositide 3-kinase (PI3K)/protein kinase B (Akt) and mitogen-activated protein kinase (MAPK) pathways, which also drive angiogenesis. This review discusses the inhibition of angiogenesis through the upregulation of PTEN and the inhibition of hypoxia-inducible factor 1 alpha (HIF-1-α) in human malignant melanoma, as no targeted therapies have been approved by the FDA for the inhibition of angiogenesis in human malignant melanoma. The emergence of nanocarrier formulations to enhance the pharmacokinetic profile of phytochemicals that upregulate PTEN activity and improve the upregulation of PTEN has also been discussed.

## 1. Introduction

Cancer is one of the leading causes of death worldwide, relative to strokes and coronary heart disease. An estimated 19.3 million new cancer cases and 10 million new cancer deaths were recorded in 2020. Furthermore, the global cancer burden has been predicted to increase by 47% from 2020 to 2040, highlighting the significance of developing new therapeutics in addition to the existing therapeutics to treat cancer [1]. Melanoma is the most lethal form of skin cancer and accounts for the majority of skin cancer-related deaths [2]. Melanoma can be classified as cutaneous melanoma, acral melanoma, mucosal melanoma, and uveal melanoma, which is dependent on where the melanoma arises from [3]. Cutaneous melanoma is the most common form of melanoma, with various subtypes such as superficial spreading melanoma (SSM), nodular melanoma (NM), lentigo maligna melanoma (LMM), amelanotic melanoma (AM), and acral lentiginous melanoma (ALM) [4]. Superficial spreading melanoma is regarded as the most common subtype, accounting for 70% of cases, and the BRAF^V600E^ mutation is present in 66% of SSM in comparison to other subtypes [2,5]. Melanoma is predominantly driven by the deregulation of two key signaling pathways: the mitogen-activated protein kinase (MAPK) and the Phosphatidylinositol-3-kinase (PI3K) pathway [6]. The main driver mutation in melanoma is BRAF^V600E^, and the downregulation of phosphatase tensin homolog (PTEN) leads to deregulation of key signaling pathways that also lead to enhanced expression of anti-apoptotic proteins [6,7,8,9]. The traditional treatment modalities for cancer are surgery, chemotherapy, radiation therapy, and surgery followed by radiation therapy, which is effective for benign tumors or early-stage cancer; however, for certain solid cancers, targeted therapies and immunotherapies have proven to be more effective [10]. Several targeted angiogenesis inhibitors, approved by the US Food and Drug Administration (FDA), have been developed, such as Bevacizumab (a monoclonal antibody that targets vascular endothelial growth factor (VEGF), which has proven to be effective for the treatment of solid cancers such as metastatic colorectal cancer [11]. The phosphatase tensin homolog (PTEN) tumor suppressor is a key regulator of angiogenesis through hypoxia-inducible factor alpha (HIF-1-α) [12,13,14,15]. Thus, the upregulation of PTEN activity may be effective for treating solid cancers driven by angiogenesis and PTEN mutations [16]. Furthermore, to improve the bioavailability of phytochemicals that upregulate PTEN activity, nanotechnology has come to the fore with the development of nanomedicines, which are effective drug delivery systems [17]. Certain nanocarrier formulations have been approved by the FDA for the treatment of various cancers [18]. The review highlights the inhibition of angiogenesis through the upregulation of PTEN in human malignant melanoma, as no targeted therapeutics have been approved by the FDA as angiogenesis inhibitors for human malignant melanoma. The upregulation of PTEN activity using phytochemicals and the use of nanocarrier formulations to improve the pharmacokinetic profile and percentage upregulation of PTEN by some phytochemicals were also discussed.

## 2. The Tumor Suppressor Phosphatase Tensin Homolog (PTEN)

**Figure 1:**
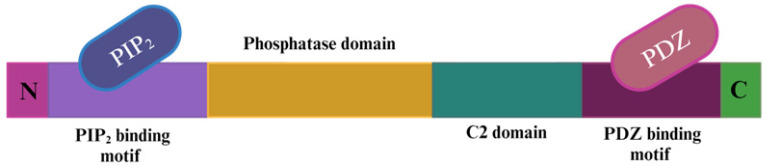
Depiction of the phosphatase tensin homolog (PTEN) gene, comprised of the N-terminal domain, PIP_2_ binding motif, phosphatase domain, C2 domain, PDZ binding motif, and C-terminal domain.

The PTEN gene is located on the human chromosome at 10q23.3 and comprises a phosphatidylinositol 4,5-bisphosphate (PIP_2_) binding site at the N-terminus, a phosphatase domain, a C2 domain, and a PDZ binding motif at the C-terminus (Figure 1) [19]. Phosphatase tensin homolog (PTEN) serves as a dual phosphatase on lipids and proteins, and its lipid phosphatase functionality regulates the phosphatidylinositol-3- kinase (PI3K)/protein kinase B (Akt)/mechanistic target of the rapamycin (mTOR) pathway. The regulation of the PI3K pathway occurs through the dephosphorylation of phosphatidylinositol 3,4,5-triphosphate (PIP_3_) to PIP_2_. The dephosphorylation of PIP_3_ ensures that downstream kinases such as phosphatidylinositol-dependent kinase 1 (PDK1) and Akt are not activated, as Akt leads to the constitutive activation of the cell cycle through the inactivation of inhibitor proteins, such as cyclin-dependent kinase inhibitor 1 (p21cip1) [20], the expression of anti-apoptotic proteins, angiogenesis, enhanced metabolism, and migration [21]. Germ-line mutations that affect the phosphatase domain of the PTEN gene can lead to diseases such as Cowden syndrome, Bannayan–Riley–Ruvalcaba syndrome, Proteus syndrome, and Proteus-like syndrome (PTEN Hamartoma syndromes) that predispose individuals to cancer [22]. Alterations in the PTEN gene have also accounted for thyroid, liver, glioblastoma, breast, melanoma, prostate, and lung cancers [23]. In metastatic melanoma, PTEN mutations display a mutation rate of 30–40% and 10% in primary melanomas, highlighting the significant role of PTEN mutations in the development of metastatic melanoma [24].

## 3. Transcriptional, Epigenetic, and Post-Translational Modifications of PTEN

Phosphatase tensin homolog (PTEN) expression can be regulated at the transcriptional level and through epigenetic mechanisms such as methylation and acetylation. There are several transcription factor binding sites at the PTEN promoter, and the binding of transcription factors either activates or represses PTEN activity. Transcription factors such as p53 and peroxisome proliferator activated receptor (PPAR)-γ activate PTEN transcription, whereas zinc finger protein (SNAI1 or SNAIL), the inhibitor of DNA-binding protein ID1, inhibits PTEN transcription. PTEN activity can also be regulated by various post-translational modifications, i.e., interaction of microRNA-25 (miR25) with PTEN, ubiquitylation, phosphorylation, oxidation, S-nitrosylation, acetylation, and sumoylation, resulting in the inhibition of PTEN lipid phosphatase activity and leading to the constitutive activation of the PI3K/Akt/mTOR pathway [25]. DNA methylation and acetylation are predominant epigenetic modifications of the PTEN gene, and DNA methylation is a well-known mechanism of gene silencing by inhibiting transcription. The process of DNA methylation involves the modification of DNA-by-DNA methyltransferases (DNMT), which covalently link methyl groups from S-adenosyl-L- methionine (SAM) to cytosine in CpG islands. Areas of the gene, such as promoters and repeated elements, harbor CpG islands. Erroneous DNA methylation can interfere with the lipid phosphatase activity of PTEN, subsequently affecting the cell cycle, cell proliferation, angiogenesis, and apoptosis [26]. To confirm the role of hypermethylation in the downregulation of PTEN activity, positional methylation of CpG-3 within the promoter of the PTEN gene in metastatic melanoma and primary melanoma cells (harboring wildtype PTEN) was conducted. The normal human melanocytes (NHM) and human malignant melanoma cell line (A375) displayed methylation rates lower than 10%, whereas melanoma cell lines harboring a PTEN mutation (SKMEL28 and MV3) displayed high methylation rates of between 58% and 91%. Furthermore, the MV3 cells were treated with 3 and 10 µmol/L of 5-azacytidine (a demethylating agent) for 4 days, and western blotting was conducted to determine whether the downstream kinase Akt would be inhibited. The substrate of Akt, GSK3, displayed reduced phosphorylation, confirming that methylation does indeed regulate PTEN activity [27].

Ubiquitylation is a post-translational modification that alters PTEN activity, and in melanoma, the neural precursor cell expressed by the developmentally downregulated gene 4-1 (NEDD4-1) E3 ligase forms a covalent bond with lysine^11^ and lysine^48^ of the PTEN promoter, resulting in the inactivation of PTEN activity through proteasomal degradation [28]. Furthermore, phosphorylation of amino acids, threonine^366^, serine^370^, threonine^382^, threonine^383^, and serine^385^, in the C-terminal region of the PTEN gene results in a more closed and stable conformation that reduces the association of PTEN with membrane phospholipids such as PIP_3_ [29]. An increase in reactive oxygen species can lead to the oxidation of the active cysteine^124^ site, leading to the formation of an intramolecular disulfide bond with cysteine^71^, thereby repressing the lipid phosphatase activity of PTEN. However, the physical interaction of PTEN and peroxiredoxin-1 prevents the formation of a disulfide bond [30]. In addition, S-nitrosylation is triggered by an increase in nitric oxide (NO), leading to the suppression of PTEN activity and the sumoylation of PTEN at lysine^266^ in the C2 domain [31]. This enhances affinity to the plasma membrane, thereby enhancing the localization of PTEN, which in turn leads to the suppression of PI3K-Akt signaling [32]. Lastly, in response to growth factor stimulation, histone acetyltransferase (PCAF) interacts with PTEN and triggers acetylation at lysine^125^ and lysine^128^ in the phosphatase domain of PTEN, resulting in the inactivation of PTEN activity [33].

## 4. What Is Angiogenesis?

**Figure 2:**
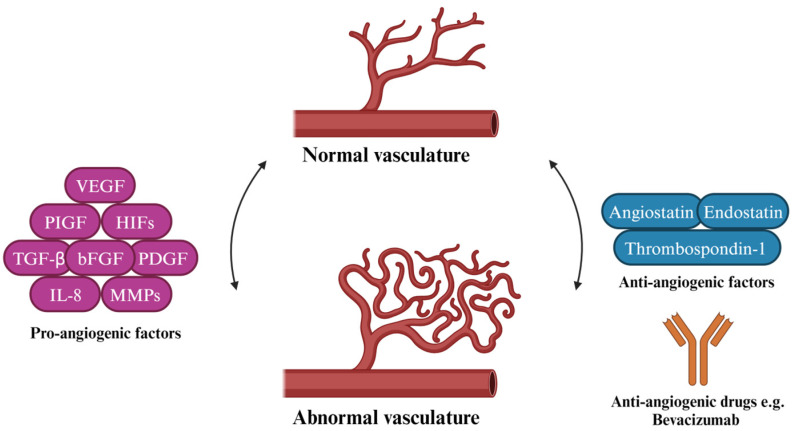
Depiction of the pro-angiogenic factors that lead to the transition from normal vasculature to abnormal vasculature. Anti-angiogenic factors and anti-angiogenic drugs (Bevacizumab) revert abnormal vasculature to normal vasculature.

Angiogenesis is the formation of new vessels from pre-existing blood or lymphatic vasculature and is a physiological process often activated for embryogenesis, wound healing, and during the menstrual cycle [34]. Although the balance between pro- and anti-angiogenic factors is tightly regulated, this balance can be offset, resulting in what is termed the angiogenic switch [35]. The angiogenic switch occurs due to rapid cellular division during tumor growth, which increases oxygen demand, leading to localized hypoxia in the tumor microenvironment and resulting in the secretion of pro-angiogenic factors [36] (Table 1). This pro-angiogenic environment enables cancer cells to metastasize to distant sites, resulting in enhanced malignancy [37]. Melanoma, in the vertical growth phase, makes use of angiogenesis to metastasize to distant sites, thereby enhancing the aggressiveness of melanoma. However, under normal and tightly regulated physiological conditions, tumors would typically only grow from 1–2 mm^3^ without the supply of nutrients and oxygen, but in a pro-angiogenic environment, the tumor can grow beyond the 1–2 mm^3^ threshold [38] (Figure 2).

## 5. FDA-Approved Drugs for the Treatment of Angiogenesis

The tumor microenvironment is skewed towards a more pro-angiogenic environment where VEGF is secreted by cancer cells and subsequently binds to vascular endothelial growth factor receptor-1 or -2 (VEGFR-1 or -2), thereby enabling the proliferation and maturation of endothelial cells. The integrins and proteins that enable the movement of endothelial cells are also activated, resulting in the migration of endothelial cells to cancer cells, thereby enabling the formation of blood vessels that supply tumors with oxygen and nutrients. Various drugs have been approved by the FDA that either target VEGF directly, target VEGFR, or other receptors that stimulate angiogenesis (Table 2).

## 6. The Link between PTEN and Angiogenesis

The tumor vasculature in melanoma is hypoxic in comparison to the oxygen pressure in other organs, thus, the expression of HIF-1-α is stabilized. In normoxic cells, HIF-1-α is degraded as it is stabilized by PTEN and conjugated to ubiquitin for proteasomal degradation. In hypoxic cells, where PTEN is inactive, HIF-1-α enters the nucleus and forms a dimer with HIF-1-β, thereby forming a transcription factor. The HIF-1-α/HIF-1-β heterodimer and the co-activator CBP/p300 subsequently bind to the hypoxia response element (HRE) on various genes, including VEGF mRNA, resulting in increased VEGF levels [15,69]. The enhanced expression of VEGF leads to increased angiogenesis either through the recruitment of endothelial progenitor cells from bone marrow cells to the tumor vasculature or through autocrine loops in the tumor cell where VEGF binds to VEGFR-1 or VEGFR-2 on tumor cells [70], resulting in the expression of downstream proteins that enhance the survival, proliferation, and vascular permeability of the tumor [71].

## 7. Phytochemicals That Display Anti-Angiogenic Activity through the Upregulation of PTEN Activity

Epigenetic modifications such as methylation and acetylation, as well as microRNAs such as microRNA 21 (miR21), inactivate the PTEN gene, however, phytochemicals such as red raspberry extract, resveratrol, curcumin, sulforaphane, and genistein have displayed demethylating effects, resulting in the upregulation of PTEN activity and the subsequent downregulation of the PI3K/Akt pathway and angiogenesis. Red raspberry extract enhanced PTEN activity through demethylating the PTEN gene promoter and inhibiting DNA methyltransferase-1 (DNMT1) expression, thus decreasing Akt activation in hepatocellular carcinoma cells [72]. Resveratrol, combined with Vitamin D3, was effective in the demethylation of the PTEN promoter, downregulating DNMT in the breast cancer cell line (MCF-7) [73]. Difluorinated curcumin, an analogue of curcumin isolated from *Curcuma longa* L., displayed antiproliferative activity against 5-fluorouracil (5-FU) + Oxaliplatin-resistant colon cancer cells, downregulated miR21 in chemo-resistant colon cancer (HCT116 and HT-29) cells, and restored PTEN function [74]. Furthermore, indole-3-carbinol, isolated from cruciferous vegetables such as broccoli and Brussels sprouts, at 200 µM displayed a 2-fold increase in the PTEN protein levels of G361 melanoma cells, whereas a 10-fold increase in PTEN protein levels was detected in SKMEL-30 melanoma cells [28]. Thymoquinone, isolated from *Nigella sativa* L., at 50 µM upregulated PTEN mRNA in doxorubicin-resistant breast cancer (MCF-7/DOX) cells by 7.9-fold after 24 h [75]. Thus, it can be hypothesized that, through the restoration of PTEN activity, HIF-1-α will be ubiquitylated by PTEN and undergo proteasomal degradation, resulting in decreased angiogenesis (Table 3).

## 8. Enhancing the Efficacy of Phytochemicals Used to Upregulate PTEN Activity Using Nanocarriers

Phytochemicals derived from plants such as resveratrol, curcumin, genistein and sulforaphane have immense pharmacological properties that have been explored for the treatment of various diseases, particularly cancer [81,82,83,84]. Although the use of phytochemicals for the treatment of cancer has grown in popularity, the low bioavailability and solubility of most phytochemicals limit their clinical use [85]. To overcome these obstacles, nanocarrier formulations could be used [86]. The isolation of paclitaxel from *Taxus brevifolia* Nutt. (Pacific yew) or *Taxus baccata* L. (English yew) for the treatment of cancer was one of the most important isolated phytochemicals. Taxol (commercial formulation) was approved by the FDA in 1992 for the treatment of ovarian cancer but is now used for the treatment of other cancers as well [87]. Paclitaxel displays limitations such as low water solubility, thus, encapsulation of paclitaxel in lipid-based nanocarriers enhances the solubility and efficacy of paclitaxel against various cancers, such as ovarian, metastatic breast, and non-small cell lung cancer [88]. Furthermore, the efficacy of thymoquinone is limited in vivo due to poor biological stability, a short half-life, hydrophobicity and low bioavailability [89]. To enhance the efficacy of thymoquinone in vivo, various nanocarrier formulations have been developed using inorganic and organic materials [89]. Notably, thymoquinone liposomes (TQ-LP) and free thymoquinone (TQ) displayed antiproliferative activity against two human metastatic breast cancer cell lines (T-47D and MCF-7). TQ-LP displayed a 50% effective dose (ED_50_) of 75 µM and 200 µM, respectively, whereas the ED_50_ values of TQ were 15 µM and 40 µM, respectively, against the two breast cancer cell lines. The TQ-LP displayed an ED_50_ of 350 µM on periodontal ligament fibroblasts (normal cells), whereas the TQ displayed an ED_50_ of 85 µM [90]. There are no reports of nanocarriers encapsulated/conjugated with thymoquinone displaying upregulation of PTEN activity (Table 4). Furthermore, nanocarriers for the delivery of drugs have gained traction, thus, the FDA has approved several nanocarrier formulations that have been shown to be more effective than traditional chemotherapeutics with fewer off-target effects (Table 5).

## 9. Discussion

Human malignant melanoma (HMM) is the most lethal form of skin cancer, and several therapeutics have been developed, such as the chemotherapeutic agent Dacarbazine, which was approved by the FDA in 1974 for HMM [95]. In addition to chemotherapeutics, therapeutics that target the different hallmarks of cancer, such as angiogenesis, which is a key driver of HMM, have been explored [96]. The inhibition of VEGF (the main pro-angiogenic factor of melanoma) was identified as an alternative approach for the treatment of HMM, and several anti-angiogenic drugs have been approved by the FDA for the treatment of various solid cancers (Table 2). However, no anti-angiogenic drugs have been approved for HMM [29]. Several clinical trials have been conducted with Bevacizumab (a monoclonal antibody that inhibits VEGF) as a monotherapy, and in one trial, the median overall survival was 9 months, however the combination of Bevacizumab and an immune checkpoint inhibitor (ipilimumab) displayed an overall survival rate of 25.1 months, highlighting the efficacy of combination therapies for melanoma in comparison to monotherapies [96,97]. Furthermore, 70% of patients with melanoma displayed mutations in genes in key signaling pathways such as PTEN, which leads to the hyperactivation of the PI3K/Akt/mTOR pathway [95]. Several inhibitors have been developed for the inhibition of the PI3K/Akt/mTOR pathway, however the upregulation of PTEN activity is being explored as an alternative approach that may also aid in the inhibition of angiogenesis. For the treatment of ovarian cancer, this phenomenon was investigated in an in vitro study, where the polyphenol gallic acid at 40 µM inhibited VEGF in A2780/CP70 and OVCAR-3 cells by 24.05 and 27.12%, respectively [98]. Furthermore, gallic acid at 20 µM downregulated HIF-1-α activity to 23.48% [98]. To confirm that the inhibition of VEGF was due to the downregulation of HIF-1-α, a VEGF promoter reporter and HIF-1-α plasmids were transfected into OVCAR-3 cells, and the inhibition of VEGF was reversed due to the constitutive expression of HIF-1-α, confirming that angiogenesis in ovarian cancer cell lines is dependent on the expression of HIF-1-α [98]. This study also highlighted the upregulation of PTEN activity and it was hypothesized that the upregulation of PTEN resulted in the inhibition of HIF-1-α [98]. Furthermore, Isoliquiritigenin (ISL) isolated from the roots of *Glycyrrhiza uralensis* significantly inhibited VEGF-induced tube formation and sprout formation (chick aortic ring model) after 48 h at 20 µM [106,107]. Wang et al. also displayed that ISL inhibited HIF-1-α protein expression dose-dependently in breast cancer cells (MDA-MB-231), thus validating the anti-angiogenic effects [107]. Peng et al. further displayed through quantitative polymerase chain reaction (qPCR) the inhibition of microRNA-374a (miR-374a) messenger RNA (mRNA) by ISL dose-dependently and upregulation of PTEN mRNA 15-fold in breast cancer cells (MCF-7 and MDA-MB-231) after 24 h [108]. Thus, the inhibition of HIF-1-α and subsequent downregulation of angiogenesis could be linked to the upregulation of PTEN activity. Li et al. displayed, through western blotting, the significant dose-dependent reduction of HIF-1-α and VEGF by triptolide (isolated from *Tripterygium wilfordii*) in osteosarcoma cells (MG-63) [106,109]. In another study, Li et al. displayed the downregulation of microRNA-21 (miR-21) in non-small cell lung cancer cells (NSCLC, PC-9) by triptolide at 25 and 50 nM [110]. Through western blotting, PC-9 cells pre-treated with 25 and 50 nM triptolide enhanced PTEN protein expression [110]. Thus, highlighting the hypothesized link between PTEN and angiogenesis, both studies highlighted the inhibition of HIF-1-α and VEGF and the upregulation of PTEN protein expression. In another study, Wang et al., displayed, through western blotting, the inhibition of HIF-1-α protein expression in U87 gliomas and inhibition of VEGF at 40 mg/kg by baicalein isolated from the roots of *Scutellaria baicalensis* [106,111]. Baicalein enhanced PTEN expression in NSCLCs (A549 and H460) at 40 and 80 µmol/L through western blotting [112]. Celastrol isolated from *Tripterygium wilfordii* at 0.75 to 2 µg/mL inhibited the migration of endothelial cells (EA.hy926) with an IC_50_ of 1.35 µg/mL, thus, Celastrol inhibited the hypoxia-induced migration of endothelial cells [106,113]. In hepatocellular carcinoma (HepG2) cells, Celastrol at 2 and 4 µg/mL inhibited nuclear HIF-1-α protein expression through western blotting and Celastrol also inhibited HIF-1-α mRNA dose-dependently in HepG2 and A549 cells [113]. Huang et al. further investigated the effect of Celastrol on VEGF mRNA through real-time polymerase chain reaction (RT-PCR), and Celastrol dose-dependently inhibited VEGF mRNA expression [113]. Zhu et al. displayed enhanced PTEN expression dose-dependently by Celastrol in cholangiocarcinoma cells (TFK-1) [114]. The upregulation of PTEN activity in HMM using phytochemicals that target factors that downregulate PTEN activity has been extensively highlighted, however no studies have highlighted the inhibition of angiogenesis through the upregulation of PTEN activity and inhibition of HIF-1-α in HMM. This study also highlights a gap in research in terms of anti-angiogenic therapeutics due to the development of resistance. The two key signaling pathways (MAPK and PI3K pathways) that largely account for melanoma tumorigenesis are regulated by PTEN [98]. PTEN’s lipid phosphatase activity regulates the PI3K pathway, and PTEN’s protein phosphatase activity regulates the MAPK pathway through the dephosphorylation of focal adhesion kinase (FAK), thereby inhibiting angiogenesis [99]. This highlights the need for further research and development of therapeutics that upregulate PTEN activity and inhibit angiogenesis [99]. Furthermore, several phytochemicals have been shown to upregulate PTEN activity, however due to their low solubility, stability, bioavailability and target specificity, their use in the treatment regimen for cancer is limited [100]. Thus, nanocarrier formulations have proven to increase the solubility and stability, reduce premature degradation, and increase the circulation time of some phytochemicals in the body [100]. The use of chemotherapeutic drugs and phytochemicals for the treatment of cancer has also largely been impacted by the development of resistance, resulting in the need for the administration of higher doses, which may lead to severe adverse effects similar to vincristine (Table 3) but through the encapsulation of vincristine in a nanocarrier formulation (Marqibo), the neurotoxicity was comparable to the neurotoxicity displayed by vincristine at lower doses, thereby showing a decrease in toxicity. Nanocarrier formulations encapsulated with phytochemicals also displayed enhanced biological activity, which was evident when the viability of A549 lung cancer cells was reduced by 60% and 100% by quercetin and quercetin nanomicelles at 100 µM [101]. Furthermore, through the chick chorioallantoic membrane (CAM) assay, epigallocatechin-3-gallate nanoparticles at 3 µg and epicallocatechin-3-gallate at 35 µg inhibited angiogenesis by 57% and 35%, respectively, highlighting the increased anti-angiogenic activity displayed by the nanoformulation of epigallocatechin-3-gallate [102]. These studies emphasize the improved pharmacokinetic profile, biological activity and limited toxicity of nanocarrier formulations compared to the free drug. Furthermore, no studies display the upregulation of PTEN activity using phytochemical nanocarrier formulations for HMM. Thus, more studies need to be conducted to determine whether phytochemicals such as thymoquinone (upregulates PTEN activity in melanoma) will display enhanced upregulation of PTEN activity when encapsulated in nanocarrier formulations.

## 10. Conclusions

Angiogenesis plays an imperative role in the tumorigenesis of some solid tumors, thus, several anti-angiogenic drugs have been developed to target tumor angiogenesis. The targeted therapies for angiogenesis often acquire resistance due to other pro-angiogenic factors present in the tumor microenvironment, and there are several adverse effects when using anti-angiogenic drugs. Due to the adverse effects, lower doses are prescribed, limiting the efficacy of the drug. Thus, this review elucidates the link between PTEN and angiogenesis through HIF-1-α and highlights that instead of directly targeting tumor angiogenesis, the tumor suppressor, PTEN, could be upregulated using phytochemicals, resulting in the ubiquitylation of HIF-1-α and a reduction in angiogenesis. Furthermore, the pharmacokinetic profile of phytochemicals can be improved using nanocarrier formulations. The FDA has approved a few nanocarrier formulations; however, more research needs to be conducted to elucidate the in vivo toxicities that may arise with some of the nanocarrier formulations.

## Figures and Tables

**Table 1:** Pro-angiogenic and anti-angiogenic factors of melanoma.

**Pro-Angiogenic Factors**	**Function**	**Reference**
Chemokines (CXCL)-1, -2, -3, -5, -6, -7, -8	Chemokines are structurally related cytokines that play a crucial role in inflammation, immunity, and angiogenesis. CC and CXCL chemokines both play an integral role in tumor angiogenesis, which is required for sustained tumor proliferation. The CXCL chemokines, identified by the presence of the glutamic-leucine-arginine (ELR) motif at the N-terminal, can be divided into ELR+ chemokines that promote angiogenesis and ELR− chemokines that suppress angiogenesis or have angiostatic effects.	[31]
Vascular endothelial growth factor (VEGF)-A, -B, -C, -D, E and placenta growth factor (PlGF)-1 and -2	The VEGF gene secretes six glycoproteins, namely: VEGF-A, -B, -C, -D, -E, and placenta growth factor (PlGF)-1 and -2. The predominant glycoprotein associated with tumor angiogenesis is VEGF-A, which can give rise to five isoforms through mRNA alternative splicing, namely VEGF111, VEGF121, VEGF165, VEGF189, and VEGF206. The predominant isoform, VEGF165, is overexpressed by several malignant tumors, such as malignant melanoma, and the expression of isoforms is tissue-specific; thus, these isoforms play pertinent roles in vasculogenesis and tumor angiogenesis. The binding of the glycoprotein VEGF-A to its receptor, VEGFR1, triggers the activation of several signaling pathways that ensure sustained endothelial cell survival, mitogenesis, migration, differentiation, vascular permeability, and recruitment of endothelial progenitor cells from the bone marrow to the tumor vasculature.	[31]
Basic fibroblast growth factor (bFGF)	Stimulates angiogenesis in melanoma through the upregulation of VEGF and matrix metalloproteinase (MMP) expression and promotes endothelial cell proliferation and migration.	[31,32]
HIF-1, -2, -3	The predominant expression of hypoxia-inducible factors (HIFs) occurs due to the stabilization of hypoxia when tumors outgrow their vascular network. Furthermore, stabilization of hypoxia leads to the transcription of genes that promote enhanced angiogenesis, energy metabolism, cell survival, radiation resistance, invasion, and metastasis.	[31,32]
Platelet-derived growth factor (PDGF)-A, -B, -C, -D	Plays a role in the autocrine stimulation of cancer as it directly stimulates the growth of cancer cells. Additionally, it plays a role in the paracrine stimulation of cancer as it indirectly stimulates carcinogenesis through the stimulation of angiogenesis.	[31]
Transforming growth factor beta (TGF-β)-1, -2, -3	Transforming growth factor beta (TGF-β) is a cytokine involved in the promotion of carcinogenesis through various hallmarks of cancer, such as the evasion of apoptosis and stimulation of angiogenesis. The TGF-β family comprises three isoforms: TGF-β-1, -2 and -3, where TGF-β-1 is the predominant isoform linked to the stimulation of VEGF, which in turn directly stimulates angiogenesis in melanoma.	[31]
Interleukin-8 (IL-8)	Tumor-derived IL-8 promotes angiogenesis, tumor proliferation, and migration in melanoma. Similarly, IL-8 derived from endothelial cells promotes the migration of tumor cells.	[31,32]
Matrix metalloproteinases (MMP-2, -3, -7, -9 and -14)	Proteases are involved in bone resorption, wound healing, and angiogenesis. The MMPs produced by tumor cells facilitate angiogenesis, tumor growth, and metastasis. MMP-2 and MMP-9 are the main drivers of angiogenesis in melanoma through the degradation of the extracellular matrix (ECM) and the activation of VEGF and TGF-β.	[31,32]
**Antiangiogenic factors**	**Function**	**Reference**
Angiostatin	Angiostatin formation occurs through the proteolytic digestion of plasminogen. Angiostatin selectively inhibits endothelial cell proliferation after administration of serum/urine from tumor-bearing mice. Furthermore, in human prostate carcinoma cells (PC-3), the release of urokinase (uPA) and free sulfhydryl donors (FSDs) generates angiostatin from plasminogen, and non-cell-derived angiostatin suppresses angiogenesis in vitro and in vivo, highlighting the potential use of recombinant angiostatin as an anti-angiogenic therapeutic/drug.	[31,32,33]
Endostatin	The cleavage of collagen XVIII, an important component of the basement membrane in the extracellular matrix, results in the formation of a 20 kda C—terminal fragment, which possesses anti-angiogenic activity. The anti-angiogenic activity is elicited by inhibiting proliferation, migration, and enhancing apoptosis in endothelial cells. Endostatin also suppresses angiogenesis through the competitive inhibition of VEGFR1 and VEGFR2, thereby enhancing the production of thrombospondin-1, which in turn suppresses angiogenesis.	[31,32]
Thrombospondin (TSP)-1	Thrombospondins are a family of extracellular matrix (ECM) proteins, predominantly found in embryonic and adult tissues, consisting of five members: TSP1, TSP2, TSP3, TSP4, and TSP5. Thrombospondin-1 has been identified as an endogenous inhibitor of angiogenesis through the inhibition of endothelial cell migration, proliferation, and induction of apoptosis in endothelial cells. Thrombospondin-1 also elicits anti-angiogenic activity through the inhibition of VEGFR2 through the ligation of its receptor (CD47).	[31,32]

**Table 2:** Anti-angiogenic drugs approved by the FDA for various cancers.

Therapy	Mechanism	Side-Effects	Cancer	References
Axitinib (Inlyta)	Selectively inhibits in vitro VEGFR-1, -2 and -3 at sub-nanomolar concentrations. In in vivo pre-clinical models, it demonstrated anti-angiogenic activity through the modulation of VEGFR-1, -2 and -3.	Hypertension, diarrhoea, nausea, hand-foot syndrome, fatigue, and hypothyroidism	Advanced Renal cell carcinoma	[39,40,41]
Bevacizumab (Avastin^®^)	Monoclonal antibody that neutralizes circulating VEGF such that VEGF cannot bind the tyrosine kinase receptors (VEGFR-1, -2 and -3). This results in the lowering of interstitial pressure, thereby increasing in vascular permeability and enhancing the delivery of chemotherapeutic agents.	Hypertension, proteinuria, epistaxis, thrombosis, and gastrointestinal bleeding	Metastatic colorectal cancer	[42,43,44]
Cabozantinib (Cometriq^®^)	A small molecule inhibitor that inhibits multiple tyrosine kinase receptors implicated in the pathogenesis of medullary thyroid cancer i.e., RET, MET and VEGFR-2	Gastrointestinal perforation, hemorrhage, hypertension, and venous thrombosis	Progressive, unresectable locally advanced or metastatic medullary thyroid cancer	[45,46]
Everolimus (Afinitor^®^)	Inhibits mTORC2 linked to the PI3K and VEGF pathway. It is used concurrently with drugs such as sorafenib and sunitinib that inhibit angiogenesis	Stomatitis, Asthenia, fatigue, rash, diarrhea, nausea, mucosal inflammation, oedema peripheral, infections, dyspnea, pneumonitis, anemia, lymphopenia, thrombocytopenia, hypercholesterolemia, hypertriglyceridemia, hyperglycemia, and elevated creatinine	Clear cell metastatic renal cell cancer	[47,48,49]
Lenalidomide (Revlimid^®^)	Inhibits angiogenesis through the inhibition of pertinent angiogenic factors i.e., VEGF, bFGF and HIF. Lenalidomide also inhibits endothelial cell migration, adhesion, capillary tube formation and endothelial cell apoptosis in 3D collagen cultures.	Neutropenia, thrombocytopenia, anemia, infections, and thrombosis	Multiple myeloma	[16,50,51,52]
Lenvatinib mesylate (Lenvima^®^)	Inhibits angiogenesis through the inhibition of multiple tyrosine kinase receptors, namely VEGFR1-3, FGFR1-4, RET, c-KIT, and PDGFRβ.	Hypertension, diarrhea, fatigue or asthenia, appetite loss, weight loss and nausea	Progressiveradioiodine-refractory differentiated thyroid cancer	[53,54]
Pazopanib (Votrient^®^)	Anti-angiogenic activity through the inhibition of VEGFR1-3 and PDGFR-α^28^ and PDGFR-β on endothelial cells. Inhibition of tyrosine kinase receptors results in the inhibition of pathways that promote cell proliferation, cell survival, vascular permeability, and cell migration.	Diarrhea, hypertension, and elevation of liver enzymes	Metastatic renal cell carcinoma	[55,56]
Ramucirumab (Cyramza^®^)	Anti-angiogenic activity through the inhibition of VEGFR-2 on endothelial cells. The inhibition of VEGFR-2 enables the inhibition of signaling pathways in endothelial cells that promote cell proliferation, cell survival, increased vascular permeability and differentiation.	Fatigue, abdominal pain, appetite loss, vomiting, constipation, anemia, dysphagia, hypertension, hemorrhage, arterial thromboembolism, venous thromboembolism, proteinuria, gastrointestinal perforation, fistula formation, infusion-related reaction, and cardiac failure	Advanced gastric cancer or gastro-esophageal junction adenocarcinoma	[57,58]
Regorafenib (Stivarga^®^)	Orally active, diphenylurea multikinase inhibitor of VEGFR1-3, c-KIT, TIE-2, PDGFR-β, FGFR-1, RET, RAF-1, BRAF and p38 MAP kinase. The inhibition of several tyrosine kinase receptors leads to the inhibition of angiogenesis and oncogenesis	Hand-foot skin reaction, rash, desquamation, alopecia, fatigue, hypertension, mucotitis, diarrhea, and thyroid dysfunction	Metastatic colorectal cancer	[59,60]
Sorafenib (Nexavar^®^)	Inhibits angiogenesis and oncogenesis through the inhibition of the following tyrosine kinase receptors, i.e., RAF kinase, PDGF, VEGFR 2-3, and c-KIT	Reversible skin rashes, hand-foot skin reaction, diarrhea, hypertension, and sensory neuropathic changes	Advanced renal cell carcinoma	[61,62]
Sunitinib (Sutent^®^)	Exhibits anti-tumor and anti-angiogenic effects through the inhibition of several kinases namely, PDGFR-α, PDGFR-β, VEGFR1-3, KIT, FLT3, CSF-1R, and RET	Left ventricle dysfunction, hemorrhagic events, hypertension, fatigue, diarrhea, mucositis/stomatitis, vomiting, abdominal pain, constipation, nausea, anorexia, altered taste, headache, dyspnea, cough, skin discoloration, rash, hand-foot syndrome, arthralgia, back-pain, and myalgia	Gastrointestinal stromal tumor and metastatic renal cell carcinoma	[63,64]
Vandetanib (Caprelsa^®^)	Antagonist of VEGFR-2, EGFR, and RET kinase resulting in antiangiogenic and antineoplastic activity	Diarrhea/colitis, rash, dermatitis acneiform/acne, nausea, hypertension, hypertensive crisis, accelerated hypertension, headache, fatigue, appetite loss, abdominal pain, dry skin, vomiting, asthenia, ECG QT prolonged, photosensitivity radiation, insomnia, nasopharyngitis, dyspepsia, hypocalcemia, cough, pruritus, weight loss, proteinuria, and depression	Medullary thyroid cancer	[65,66]
Ziv-aflibercept (Zaltrap^®^)	A recombinant protein that comprises of the extracellular domains from both VEGFR-1 and VEGFR-2 fused to the fc (a) region of human IgG1. Ziv-aflibercept is a pseudo receptor that binds VEGFA, VEGFB, and PlGF resulting in the inhibition of angiogenesis	Urinary tract infection, leukopenia, neutropenia, thrombocytopenia, appetite loss, dehydration, headache, hypertension, epistaxis, dysphonia, dyspnea, oropharyngeal pain, rhinorrhea, diarrhea, stomatitis, abdominal pain, hemorrhoids, rectal hemorrhage, proctalgia, palmer-plantar erythrodysesthesia syndrome, skin hyperpigmentation, proteinuria, serum creatinine increased, fatigue, asthenia, AST increased, ALT increased, and weight loss	Metastatic colorectal cancer	[67,68]

**Table 3:** Phytochemicals that display anti-angiogenic activity through the upregulation of PTEN activity.

Phytochemical	Source	Mechanism	References
Red raspberry extract	*Rubus idaeus* L. (red raspberry)	Red raspberry extract enhanced PTEN activity through demethylating the PTEN promoter and inhibiting DNA methyltransferase-1 (DNMT-1) expression, thus, decreasing Akt activation in hepatocellular carcinoma (HepG2)	[72]
Resveratrol	Grapes, apples, blueberries, plums and peanuts	Resveratrol in combination with Vitamin D3 demethylated the PTEN promoter and downregulated DNMT in breast cancer cells (MCF-7)	[73]
Curcumin	*Curcuma longa* L. (turmeric)	An analogue of curcumin (difluorinated curcumin) displayed antiproliferative activity against 5-fluorouracil (5-FU) + oxaliplatin resistant colon cancer cells, downregulated miR21 in chemo-resistant colon cancer (HCT116 and HT-29) cells, and restored PTEN function	[74]
Sulforaphane	Cruciferous vegetables such as kale (*Brassica oleracea* L. var. acephala), cauliflower (*B. oleracea* var Botrytis L.), cabbage (*B. oleracea* L. var capitata), and broccoli (*B. oleracea* var. italica)	Demethylated the PTEN promoter, thereby inhibiting the PI3K/Akt pathway and angiogenesis	[76,77]
Genistein	Soybeans, legumes, broccoli, cauliflower and sunflowers	At 1 µM, genistein upregulated PTEN expression by 1.2-fold in the metastastic breast cancer cell (Hs578t)	[78,79,80]
Indole-3-carbinol	Cruciferous vegetables such as Kale (*Brassica oleracea* L. var. acephala), cauliflower (*B. oleracea* var botrytis L.), cabbage (*B. oleracea* L. var capitata), and broccoli (*B. oleracea* var. italica)	At 200 µM, indole-3-carbinol displayed a 2-fold and 10-fold increase in PTEN protein levels of G361 and SKMEL30 melanoma cells, respectively	[28]
Thymoquinone	*Nigella sativa* L. (black cumin)	At 50 µM, thymoquinone upregulated PTEN mRNA levels in doxorubicin resistant breast cancer (MCF-7/DOX) by 7.9-fold after 24 h	[75]

**Table 4:** Enhancing the efficacy of phytochemicals used to upregulate PTEN activity using nanocarriers.

Phytochemical	Source	Limitation/s	Nanocarrier Formulation	Mechanism	Reference
Paclitaxel	*Taxus brevifolia* Nutt (Pacific Yew) and *Taxus baccata* L. (English Yew)	Low water solubility	Lipid-based nanocarrier	The encapsulation of paclitaxel in lipid-based nanocarriers enhanced solubility and efficacy of paclitaxel against various cancers such as ovarian, metastatic breast, and non-small cell lung cancer	[88]
Thymoquinone	*Nigella sativa* L. (Black cumin)	Poor biological stability, short half-life, hydrophobicity, and low bioavailability	Liposomes	The encapsulation of thymoquinone in liposomes enhanced biological stability, increased the half-life, decreased hydrophobicity and enhanced bioavailability. Thymoquinone liposomes (TQ-LP) displayed an ED_50_ of 350 µM on periodontal ligament fibroblasts (normal cells) whereas free thymoquinone displayed an ED_50_ of 85 µM, thus, TQ-LP displayed a lower antiproliferative effect on normal cells, which is favorable	[90]

**Table 5:** Nanocarrier formulations approved by the FDA.

Nanocarrier	Drugs	Name	Indications	Side Effects Compared to Free Drug	FDA Approval Date	References
Nanoparticle	Albumin-Paclitaxel (nab-paclitaxel)	Abraxane	Metastatic breast cancer	The absence of cremophor in the paclitaxel formulation results in decreased neutropenia and rapid improvement of peripheral neutropathy with albumin-paclitaxel.	2005	[91,92,93]
Pegylated liposome	Doxorubicin	Doxil	Ovarian, metastatic breast cancer, Kaposi sarcoma	Doxil also displayed increased cardiac safety, less nausea, vomiting and neutropenia but the main dose limiting side effect of Doxil is hand-foot syndrome, which leads to tenderness and peeling of the skin. This side effect limits the dose that can be given compared with doxorubicin.	1995	[91,94,95]
Liposome	Doxorubicin	Myocet	Breast cancer	Myocet displayed improved cardiac safety, less nausea, vomiting and neutropenia. Furthermore, Myocet does not cause hand-foot syndrome and may be used at the same dosing as doxorubicin in treatment regimens, enhancing efficacy.	2000	[91,95,96]
Liposome	Vincristine	Marqibo	Philadelphia chromosome-negative lymphoblastic leukemia	The main adverse effect of vincristine is neurotoxicity, which could be detected at lower doses of administration of vincristine resulting in the capping of the dose for vincristine to 1.4–2 mg/m^2^. The dose for Marqibo was not capped and more vincristine could be delivered with a similar toxicity profile to vincristine at low doses.	2012	[91,97,98]
Liposome	Cytarabine	Depocyt	Lymphomatous malignant meningitis	Arachnoiditis, neurotoxicity, cardiotoxicity, fever, cerebellar toxicity, corneal toxicity, hepato-renal insufficiency, necrotizing enterocolitis, pancreatitis, acute respiratory distress, and dermatological side effects.	1999	[99,100]
Liposome	Irinotecan	Onivyde	Pancreatic cancer	Diarrhea, nausea, vomiting, neutropenia, and febrile neutropenia	2015	[100,101]
Pegylated conjugate	L-Asparaginase	Oncaspar	Acute lymphoblastic leukemia	Venous thromboembolism, pancreatitis, and hyperglycemia	2006	[100,102]
Recombinant DNA derived cytotoxic protein	Denileukin diftitox	Ontak	Cutaneous T cell lymphoma	Acute hypersensitivity reactions, asthenia. Nausea, vomiting and dehydration	1999	[100,103]
Polymeric nanoparticles	Leuprolide acetate	Eligard	Advanced prostate cancer	Hot flushes, fatigue testicular atrophy, dizziness, gynecomastia, and nausea	2002	[100,104,105]
Trastuzumab covalently linked to DM1 via the stable thioether linker MCC	DM1	Kadcyla	HER2+ breast cancer	Nausea, fatigue, thrombocytopenia, headache, constipation, diarrhea, epistaxis	2013	[100]

## Abbreviations

5-FU: 5-fluorouracil; A549, H460 and PC-9: non-small cell lung cancer; HCT116, HT-29: human colon cancer cells; HepG2: hepatocellular carcinoma; MCF-7, MDA-MB-221, and T-47D: human breast cancer cells; MG-63: osteosarcoma cells; PC-3: human prostate carcinoma cells; MV-3, SKMEL28: human malignant melanoma cells; TFK-1: cholangiocarcinoma; U87: glioma; Akt: protein kinase B; ALM: acral lentiginous melanoma; ALT: alanine transaminase; AM: amelanotic melanoma; AST: aspartate aminotransferase; bFGF: basic fibroblast growth factor; BRAF: v-raf murine sarcoma viral oncogene homolog B1; c-KIT: tyrosine-protein kinase KIT; CSF-1R: colony stimulating factor-1 receptor; CXCL: chemokines; cys^71^: cysteine71; cys^124^: cysteine124; DNA: deoxyribonucleic acid; DNMT: DNA methyltransferase; ECM: extracellular matrix; ECG QT: electrocardiography QT interval; EGFR: epidermal growth factor receptor; FDA: Food and Drug Administration; FGFR: fibroblast growth factor receptor; FLT3: FMS-like tyrosine kinase-3; FSD: free sulfhydryl donors; GSK3: glycogen synthase kinase-3; HIF: hypoxia inducible growth factor; HIF-1-α: hypoxia inducible growth factor-1-alpha; HRE: hypoxia response element; IC_50_: 50% inhibitory concentration; IgG1: immunoglobulinG1; KIT: tyrosine protein kinase KIT; MET: N-methyl-N’-nitroso-guanidine human osteosarcoma transforming gene; LMM: lentigo maligna melanoma; mTOR: mechanistic target of rapamycin; miR21/25: microRNA21/25; miR-374a: microRNA-374a; mRNA: messenger RNA; NEDD4-1: neural precursor cell expressed developmentally downregulated gene4-1; NHM: normal human melanocytes; NM: nodular melanoma; NO: nitric oxide; p21cip1: cyclin dependent kinase inhibitor 1; p38 MAP kinase: p38 mitogen activated protein kinase; p53: tumor protein p53; PCAF: p300/CBP-associated factor; PDGF: platelet derived growth factor; PDGRFα: platelet-derived growth factor receptor alpha; PDK1: phosphatidylinositol dependent kinase 1; PI3K: phosphatidylinositol-3-kinase; PIP_2_: phosphatidylinositol (4,5)-bisphosphate; PIP_3_: phosphatidylinositol (3,4,5)-triphosphate; PlGF: placental growth factor; PPAR-γ: peroxisome proliferator activated receptor gamma; PTEN: phosphatase tensin homolog; qPCR: quantitative polymerase chain reaction; RAF: rapidly accelerated fibrosarcoma; RET: rearranged during transfection tyrosine kinase; RT-PCR: real time polymerase chain reaction; SAM: S-adenosyl-L-methionine; SNAI1: zinc finger protein; SSM: superficial spreading melanoma; TIE-2: TEK receptor tyrosine kinase; TSP: thrombospondin; uPA: urokinase; VEGF: vascular endothelial growth factor; VEGFR: vascular endothelial growth factor receptor.

## References

[1] Sung H., Ferlay J., Siegel R.L., Laversanne M., Soerjomataram I., Jemal A., Bray F. (2021). Global Cancer Statistics 2020: GLOBOCAN Estimates of Incidence and Mortality Worldwide for 36 Cancers in 185 Countries. CA Cancer J. Clin..

[2] Saginala K., Barsouk A., Aluru J.S., Rawla P., Barsouk A. (2021). Epidemiology of Melanoma. Med. Sci..

[3] Rabbie R., Ferguson P., Molina-Aguilar C., Adams D.J., Robles-Espinoza C.D. (2019). Melanoma subtypes: Genomic profiles, prognostic molecular markers and therapeutic possibilities. J. Pathol..

[4] Long G.V., Swetter S.M., Menzies A.M., Gershenwald J.E., Scolyer R.A. (2023). Cutaneous melanoma. Lancet.

[5] El Sharouni M.A., van Diest P.J., Witkamp A.J., Sigurdsson V., van Gils C.H. (2020). Subtyping Cutaneous Melanoma Matters. JNCI Cancer Spectr..

[6] Paluncic J., Kovacevic Z., Jansson P.J., Kalinowski D., Merlot A.M., Huang M.L.H., Lok H.C., Sahni S., Lane D.J.R., Richardson D.R. (2016). Roads to melanoma: Key pathways and emerging players in melanoma progression and oncogenic signaling. Biochim. Et Biophys. Acta (BBA)—Mol. Cell Res..

[7] Chen C.-Y., Chen J., He L., Stiles B.L. (2018). PTEN: Tumor Suppressor and Metabolic Regulator. Front. Endocrinol..

[8] Hannan E.J., O’Leary D.P., MacNally S.P., Kay E.W., Farrell M.A., Morris P.G., Power C.P., Hill A.D.K. (2017). The significance of BRAF V600E mutation status discordance between primary cutaneous melanoma and brain metastases: The implications for BRAF inhibitor therapy. Medicine.

[9] Waite K.A., Eng C. (2002). Protean PTEN: Form and Function. Am. J. Hum. Genet..

[10] Debela D.T., Muzazu S.G., Heraro K.D., Ndalama M.T., Mesele B.W., Haile D.C., Kitui S.K., Manyazewal T. (2021). New approaches and procedures for cancer treatment: Current perspectives. SAGE Open Med..

[11] Mousa S.A., Davis P.J., Mousa S.A., Davis P.J. (2017). Chapter 1—Angiogenesis and Anti-Angiogenesis Strategies in Cancer. Anti-Angiogenesis Strategies in Cancer Therapeutics.

[12] Abdalla A.M.E., Xiao L., Ullah M.W., Yu M., Ouyang C., Yang G. (2018). Current Challenges of Cancer Anti-angiogenic Therapy and the Promise of Nanotherapeutics. Theranostics.

[13] Dillon L.M., Miller T.W. (2014). Therapeutic targeting of cancers with loss of PTEN function. Curr. Drug Targets.

[14] Morris L.G., Chan T.A. (2015). Therapeutic targeting of tumor suppressor genes. Cancer.

[15] Wigerup C., Påhlman S., Bexell D. (2016). Therapeutic targeting of hypoxia and hypoxia-inducible factors in cancer. Pharmacol. Ther..

[16] Rodriguez S., Huynh-Do U. (2012). The Role of PTEN in Tumor Angiogenesis. J. Oncol..

[17] Murthy S.K. (2007). Nanoparticles in modern medicine: State of the art and future challenges. Int. J. Nanomed..

[18] Foulkes R., Man E., Thind J., Yeung S., Joy A., Hoskins C. (2020). The regulation of nanomaterials and nanomedicines for clinical application: Current and future perspectives. Biomater. Sci..

[19] Molinari F., Frattini M. (2013). Functions and Regulation of the PTEN Gene in Colorectal Cancer. Front. Oncol..

[20] Chang F., Lee J.T., Navolanic P.M., Steelman L.S., Shelton J.G., Blalock W.L., Franklin R.A., McCubrey J.A. (2003). Involvement of PI3K/Akt pathway in cell cycle progression, apoptosis, and neoplastic transformation: A target for cancer chemotherapy. Leukemia.

[21] Manning B.D., Cantley L.C. (2007). AKT/PKB signaling: Navigating downstream. Cell.

[22] Hobert J.A., Eng C. (2009). PTEN hamartoma tumor syndrome: An overview. Genet. Med..

[23] Aquila S., Santoro M., Caputo A., Panno M.L., Pezzi V., De Amicis F. (2020). The Tumor Suppressor PTEN as Molecular Switch Node Regulating Cell Metabolism and Autophagy: Implications in Immune System and Tumor Microenvironment. Cells.

[24] Wu H., Goel V., Haluska F.G. (2003). PTEN signaling pathways in melanoma. Oncogene.

[25] Lee Y.R., Chen M., Pandolfi P.P. (2018). The functions and regulation of the PTEN tumour suppressor: New modes and prospects. Nat. Rev. Mol. Cell Biol..

[26] Carlos-Reyes Á., López-González J.S., Meneses-Flores M., Gallardo-Rincón D., Ruíz-García E., Marchat L.A., Astudillo-de la Vega H., Hernández de la Cruz O.N., López-Camarillo C. (2019). Dietary Compounds as Epigenetic Modulating Agents in Cancer. Front. Genet..

[27] Mirmohammadsadegh A., Marini A., Nambiar S., Hassan M., Tannapfel A., Ruzicka T., Hengge U.R. (2006). Epigenetic silencing of the PTEN gene in melanoma. Cancer Res..

[28] Aronchik I., Kundu A., Quirit J.G., Firestone G.L. (2014). The antiproliferative response of indole-3-carbinol in human melanoma cells is triggered by an interaction with NEDD4-1 and disruption of wild-type PTEN degradation. Mol. Cancer Res..

[29] Kotelevets L., Trifault B., Chastre E., Scott M.G.H. (2020). Posttranslational Regulation and Conformational Plasticity of PTEN. Cold Spring Harb. Perspect. Med..

[30] Nguyen Huu T., Park J., Zhang Y., Park I., Yoon H.J., Woo H.A., Lee S.R. (2021). Redox Regulation of PTEN by Peroxiredoxins. Antioxidants.

[31] Numajiri N., Takasawa K., Nishiya T., Tanaka H., Ohno K., Hayakawa W., Asada M., Matsuda H., Azumi K., Kamata H. (2011). On–off system for PI3-kinase–Akt signaling through S-nitrosylation of phosphatase with sequence homology to tensin (PTEN). Proc. Natl. Acad. Sci. USA.

[32] González-Santamaría J., Campagna M., Ortega-Molina A., Marcos-Villar L., de la Cruz-Herrera C.F., González D., Gallego P., Lopitz-Otsoa F., Esteban M., Rodríguez M.S. (2012). Regulation of the tumor suppressor PTEN by SUMO. Cell Death Dis..

[33] Shi Y., Paluch B.E., Wang X., Jiang X. (2012). PTEN at a glance. J. Cell Sci..

[34] Yoo S.Y., Kwon S.M. (2013). Angiogenesis and its therapeutic opportunities. Mediat. Inflamm..

[35] North S., Moenner M., Bikfalvi A. (2005). Recent developments in the regulation of the angiogenic switch by cellular stress factors in tumors. Cancer Lett..

[36] Dratkiewicz E., Simiczyjew A., Mazurkiewicz J., Ziętek M., Matkowski R., Nowak D. (2021). Hypoxia and Extracellular Acidification as Drivers of Melanoma Progression and Drug Resistance. Cells.

[37] Lugano R., Ramachandran M., Dimberg A. (2020). Tumor angiogenesis: Causes, consequences, challenges and opportunities. Cell Mol. Life Sci..

[38] Hillen F., Griffioen A.W. (2007). Tumour vascularization: Sprouting angiogenesis and beyond. Cancer Metastasis Rev..

[39] Escudier B., Gore M. (2011). Axitinib for the Management of Metastatic Renal Cell Carcinoma. Drugs R D.

[40] Mittal K., Wood L.S., Rini B.I. (2012). Axitinib in Metastatic Renal Cell Carcinoma. Biol. Ther..

[41] (2020). US Food and Drug Administration. Axitinib. https://www.accessdata.fda.gov/scripts/cder/daf/index.cfm?event=overview.process&ApplNo=202324.

[42] Kazazi-Hyseni F., Beijnen J.H., Schellens J.H.M. (2010). Bevacizumab. Oncologist.

[43] Arriaga Y., Becerra C.R. (2006). Adverse Effects of Bevacizumab and Their Management in Solid Tumors. Support. Cancer Ther..

[44] (2020). US Food and Drug Administration. Bevacizumab. https://www.accessdata.fda.gov/scripts/cder/daf/index.cfm?event=overview.process&ApplNo=125085.

[45] Hoy S.M. (2014). Cabozantinib: A review of its use in patients with medullary thyroid cancer. Drugs.

[46] (2020). US Food and Drug Administration. COMETRIQ. https://www.accessdata.fda.gov/scripts/cder/daf/index.cfm?event=overview.process&ApplNo=203756.

[47] Coppin C. (2010). Everolimus: The first approved product for patients with advanced renal cell cancer after sunitinib and/or sorafenib. Biologics.

[48] Porta C., Osanto S., Ravaud A., Climent M.-A., Vaishampayan U., White D.A., Creel P., Dickow B., Fischer P., Gornell S.S. (2011). Management of adverse events associated with the use of everolimus in patients with advanced renal cell carcinoma. Eur. J. Cancer.

[49] (2020). US Food and Drug Administration. EVEROLIMUS. https://www.accessdata.fda.gov/scripts/cder/daf/index.cfm?event=overview.process&ApplNo=022334.

[50] Galustian C., Dalgleish A. (2009). Lenalidomide: A novel anticancer drug with multiple modalities. Expert. Opin. Pharmacother..

[51] (2020). US Food and Drug Administration. Lenalidomide. https://www.accessdata.fda.gov/scripts/cder/daf/index.cfm?event=overview.process&ApplNo=021880.

[52] Rodríguez A.P.G. (2011). Management of the adverse effects of lenalidomide in multiple myeloma. Adv. Ther..

[53] (2020). US Food and Drug Administration. Lenvatinib Mesylate. https://www.accessdata.fda.gov/drugsatfda_docs/appletter/2015/206947Orig1s000ltr.pdf.

[54] Lorusso L., Newbold K. (2015). Lenvatinib: A new option for the treatment of advanced iodine refractory differentiated thyroid cancer?. Future Oncol..

[55] Keisner S.V., Shah S.R. (2011). Pazopanib. Drugs.

[56] US Food and drug administration. Pazopanib 2020. https://www.accessdata.fda.gov/scripts/cder/daf/index.cfm?event=overview.process&ApplNo=215837.

[57] Fuchs C.S., Tomasek J., Yong C.J., Dumitru F., Passalacqua R., Goswami C., Safran H., dos Santos L.V., Aprile G., Ferry D.R. (2014). Ramucirumab monotherapy for previously treated advanced gastric or gastro-oesophageal junction adenocarcinoma (REGARD): An international, randomised, multicentre, placebo-controlled, phase 3 trial. Lancet.

[58] (2020). US Food and Drug Administration. Ramucirumab. https://www.accessdata.fda.gov/drugsatfda_docs/appletter/2014/125477Orig1s000ltr.pdf.

[59] Strumberg D., Schultheis B. (2012). Regorafenib for cancer. Expert. Opin. Investig. Drugs.

[60] US Food and Drug Administration. Regorafenib 2020. https://www.accessdata.fda.gov/scripts/cder/daf/index.cfm?event=overview.process&ApplNo=203085.

[61] US Food and Drug Administration. Sorafenib 2020. https://www.accessdata.fda.gov/scripts/cder/daf/index.cfm?event=overview.process&ApplNo=021923.

[62] Kane R.C., Farrell A.T., Saber H., Tang S., Williams G., Jee J.M., Liang C., Booth B., Chidambaram N., Morse D. (2006). Sorafenib for the Treatment of Advanced Renal Cell Carcinoma. Clin. Cancer Res..

[63] Izzedine H., Buhaescu I., Rixe O., Deray G. (2007). Sunitinib malate. Cancer Chemother. Pharmacol..

[64] US Food and Drug Administration. Sunitinib 2020. https://www.accessdata.fda.gov/scripts/cder/daf/index.cfm?event=overview.process&ApplNo=021938.

[65] US Food and Drug Administration. Vandetanib 2020. https://www.accessdata.fda.gov/drugsatfda_docs/appletter/2011/022405s000ltr.pdf.

[66] Commander H., Whiteside G., Perry C. (2011). Vandetanib. Drugs.

[67] US Food and Drug Administration. Ziv-Aflibercept 2020. https://www.accessdata.fda.gov/drugsatfda_docs/appletter/2012/125418Orig1s000ltr.pdf.

[68] Patel A., Sun W. (2014). Ziv-aflibercept in metastatic colorectal cancer. Biologics.

[69] Albadari N., Deng S., Li W. (2019). The transcriptional factors HIF-1 and HIF-2 and their novel inhibitors in cancer therapy. Expert. Opin. Drug Discov..

[70] Hicklin D.J., Ellis L.M. (2005). Role of the vascular endothelial growth factor pathway in tumor growth and angiogenesis. J. Clin. Oncol..

[71] Ellis L.M., Hicklin D.J. (2008). VEGF-targeted therapy: Mechanisms of anti-tumour activity. Nat. Rev. Cancer.

[72] Zhang H., Liu J., Li G., Wei J., Chen H., Zhang C., Zhao J., Wang Y., Dang S., Li X. (2018). Fresh red raspberry phytochemicals suppress the growth of hepatocellular carcinoma cells by PTEN/AKT pathway. Int. J. Biochem. Cell Biol..

[73] Stefanska B., Rudnicka K., Bednarek A., Fabianowska-Majewska K. (2010). Hypomethylation and induction of retinoic acid receptor beta 2 by concurrent action of adenosine analogues and natural compounds in breast cancer cells. Eur. J. Pharmacol..

[74] Roy S., Yu Y., Padhye S.B., Sarkar F.H., Majumdar A.P. (2013). Difluorinated-curcumin (CDF) restores PTEN expression in colon cancer cells by down-regulating miR-21. PLoS ONE.

[75] Arafa E.S.A., Zhu Q., Shah Z.I., Wani G., Barakat B.M., Racoma I., El-Mahdy M.A., Wani A.A. (2011). Thymoquinone up-regulates PTEN expression and induces apoptosis in doxorubicin-resistant human breast cancer cells. Mutat. Res..

[76] Kim D.-H., Suh J., Surh Y.-J., Na H.-K. (2017). Regulation of the tumor suppressor PTEN by natural anticancer compounds. Ann. N. Y. Acad. Sci..

[77] Lubecka-Pietruszewska K., Kaufman-Szymczyk A., Stefanska B., Cebula-Obrzut B., Smolewski P., Fabianowska-Majewska K. (2015). Sulforaphane Alone and in Combination with Clofarabine Epigenetically Regulates the Expression of DNA Methylation-Silenced Tumour Suppressor Genes in Human Breast Cancer Cells. J. Nutr. Nutr..

[78] Garbiec E., Cielecka-Piontek J., Kowalówka M., Hołubiec M., Zalewski P. (2022). Genistein—Opportunities Related to an Interesting Molecule of Natural Origin. Molecules.

[79] Sarao L., Kaur S., Malik T., Singh A., Kour J., Nayik G.A. (2022). Chapter 19—Genistein and daidzein. Nutraceuticals and Health Care.

[80] Tuli H.S., Tuorkey M.J., Thakral F., Sak K., Kumar M., Sharma A.K., Sharma U., Jain A., Aggarwal V., Bishayee A. (2019). Molecular Mechanisms of Action of Genistein in Cancer: Recent Advances. Front. Pharmacol..

[81] Kamal M.M., Akter S., Lin C.N., Nazzal S. (2020). Sulforaphane as an anticancer molecule: Mechanisms of action, synergistic effects, enhancement of drug safety, and delivery systems. Arch. Pharm. Res..

[82] Santos A.C., Pereira I., Magalhães M., Pereira-Silva M., Caldas M., Ferreira L., Figueiras A., Ribeiro A.J., Veiga F. (2019). Targeting Cancer Via Resveratrol-Loaded Nanoparticles Administration: Focusing on In Vivo Evidence. AAPS J..

[83] Spagnuolo C., Russo G.L., Orhan I.E., Habtemariam S., Daglia M., Sureda A., Nabavi S.F., Devi K.P., Loizzo M.R., Tundis R. (2015). Genistein and cancer: Current status, challenges, and future directions. Adv. Nutr..

[84] Tomeh M.A., Hadianamrei R., Zhao X. (2019). A Review of Curcumin and Its Derivatives as Anticancer Agents. Int. J. Mol. Sci..

[85] Aqil F., Munagala R., Jeyabalan J., Vadhanam M.V. (2013). Bioavailability of phytochemicals and its enhancement by drug delivery systems. Cancer Lett..

[86] Patra J.K., Das G., Fraceto L.F., Campos E.V.R., Rodriguez-Torres M.D.P., Acosta-Torres L.S., Diaz-Torres L.A., Grillo R., Swamy M.K., Sharma S. (2018). Nano based drug delivery systems: Recent developments and future prospects. J. Nanobiotechnol..

[87] Kampan N.C., Madondo M.T., McNally O.M., Quinn M., Plebanski M. (2015). Paclitaxel and Its Evolving Role in the Management of Ovarian Cancer. Biomed. Res. Int..

[88] Ma P., Mumper R.J. (2013). Paclitaxel Nano-Delivery Systems: A Comprehensive Review. J. Nanomed. Nanotechnol..

[89] Pal R.R., Rajpal V., Singh P., Saraf S.A. (2021). Recent Findings on Thymoquinone and Its Applications as a Nanocarrier for the Treatment of Cancer and Rheumatoid Arthritis. Pharmaceutics.

[90] Odeh F., Ismail S.I., Abu-Dahab R., Mahmoud I.S., Al Bawab A. (2012). Thymoquinone in liposomes: A study of loading efficiency and biological activity towards breast cancer. Drug Deliv..

[91] Danhier F., Feron O., Préat V. (2010). To exploit the tumor microenvironment: Passive and active tumor targeting of nanocarriers for anti-cancer drug delivery. J. Control. Release.

[92] Zhao M., Lei C., Yang Y., Bu X., Ma H., Gong H., Liu J., Fang X., Hu Z., Fang Q. (2015). Abraxane, the Nanoparticle Formulation of Paclitaxel Can Induce Drug Resistance by Up-Regulation of P-gp. PLoS ONE.

[93] Gradishar W.J. (2006). Albumin-bound paclitaxel: A next-generation taxane. Expert. Opin. Pharmacother..

[94] Barenholz Y. (2012). Doxil^®^—The first FDA-approved nano-drug: Lessons learned. J. Control. Release.

[95] Rafiyath S.M., Rasul M., Lee B., Wei G., Lamba G., Liu D. (2012). Comparison of safety and toxicity of liposomal doxorubicin vs. conventional anthracyclines: A meta-analysis. Exp. Hematol. Oncol..

[96] Kanwal U., Irfan Bukhari N., Ovais M., Abass N., Hussain K., Raza A. (2018). Advances in nano-delivery systems for doxorubicin: An updated insight. J. Drug Target..

[97] Silverman J.A., Deitcher S.R. (2013). Marqibo^®^ (vincristine sulfate liposome injection) improves the pharmacokinetics and pharmacodynamics of vincristine. Cancer Chemother. Pharmacol..

[98] Yang F., Jiang M., Lu M., Hu P., Wang H., Jiang J. (2018). Pharmacokinetic Behavior of Vincristine and Safety Following Intravenous Administration of Vincristine Sulfate Liposome Injection in Chinese Patients with Malignant Lymphoma. Front. Pharmacol..

[99] Doval D., Kumar Sharma S., Kumar M., Khandelwal V., Choudhary D. (2019). Cytarabine ears—A side effect of cytarabine therapy. J. Oncol. Pharm. Pract..

[100] Nirmala M.J., Kizhuveetil U., Johnson A., Balaji G., Nagarajan R., Muthuvijayan V. (2023). Cancer nanomedicine: A review of nano-therapeutics and challenges ahead. RSC Adv..

[101] Passero F.C., Grapsa D., Syrigos K.N., Saif M.W. (2016). The safety and efficacy of Onivyde (irinotecan liposome injection) for the treatment of metastatic pancreatic cancer following gemcitabine-based therapy. Expert. Rev. Anticancer. Ther..

[102] Dinndorf P.A., Gootenberg J., Cohen M.H., Keegan P., Pazdur R. (2007). FDA drug approval summary: Pegaspargase (oncaspar) for the first-line treatment of children with acute lymphoblastic leukemia (ALL). Oncologist.

[103] Walker P.L., Dang N.H. (2004). Denileukin diftitox as novel targeted therapy in non-Hodgkin’s lymphoma. Clin. J. Oncol. Nurs..

[104] Berges R. (2005). Eligard^®^: Pharmacokinetics, Effect on Testosterone and PSA Levels and Tolerability. Eur. Urol. Suppl..

[105] Chang J.I.C., Bucci J. (2016). Unusual side effect from a luteinizing hormone-releasing hormone agonist, leuprorelin, in the treatment of prostate cancer: A case report. J. Med. Case Rep..

[106] Hong M., Shi H., Wang N., Tan H.Y., Wang Q., Feng Y. (2019). Dual Effects of Chinese Herbal Medicines on Angiogenesis in Cancer and Ischemic Stroke Treatments: Role of HIF-1 Network. Front. Pharmacol..

[107] Wang Z., Wang N., Han S., Wang D., Mo S., Yu L., Huang H., Tsui K., Shen J., Chen J. (2013). Dietary Compound Isoliquiritigenin Inhibits Breast Cancer Neoangiogenesis via VEGF/VEGFR-2 Signaling Pathway. PLoS ONE.

[108] Peng F., Tang H., Liu P., Shen J., Guan X., Xie X., Gao J., Xiong L., Jia L., Chen J. (2017). Isoliquiritigenin modulates miR-374a/PTEN/Akt axis to suppress breast cancer tumorigenesis and metastasis. Sci. Rep..

[109] Li X., Lu Q., Xie W., Wang Y., Wang G. (2018). Anti-tumor effects of triptolide on angiogenesis and cell apoptosis in osteosarcoma cells by inducing autophagy via repressing Wnt/β-Catenin signaling. Biochem. Biophys. Res. Commun..

[110] Li X., Zang A., Jia Y., Zhang J., Fan W., Feng J., Duan M., Zhang L., Huo R., Jiao J. (2016). Triptolide reduces proliferation and enhances apoptosis of human non-small cell lung cancer cells through PTEN by targeting miR-21. Mol. Med. Rep..

[111] Wang F.-R., Jiang Y.-S. (2015). Effect of treatment with baicalein on the intracerebral tumor growth and survival of orthotopic glioma models. J. Neuro-Oncol..

[112] Lu C., Wang H., Chen S., Yang R., Li H., Zhang G. (2018). Baicalein inhibits cell growth and increases cisplatin sensitivity of A549 and H460 cells via miR-424-3p and targeting PTEN/PI3K/Akt pathway. J. Cell. Mol. Med..

[113] Huang L., Zhang Z., Zhang S., Ren J., Zhang R., Zeng H., Li Q., Wu G. (2011). Inhibitory action of Celastrol on hypoxia-mediated angiogenesis and metastasis via the HIF-1α pathway. Int. J. Mol. Med..

[114] Zhu B., Wei Y. (2020). Antitumor activity of celastrol by inhibition of proliferation, invasion, and migration in cholangiocarcinoma via PTEN/PI3K/Akt pathway. Cancer Med..

